# Electronics of Anion Hot Injection-Synthesized Te-Functionalized Kesterite Nanomaterial

**DOI:** 10.3390/nano11030794

**Published:** 2021-03-19

**Authors:** Kelechi C. Nwambaekwe, Milua Masikini, Penny Mathumba, Morongwa E. Ramoroka, Samantha Duoman, Vivian Suru John-Denk, Emmanuel I. Iwuoha

**Affiliations:** SensorLab (University of the Western Cape Sensor Laboratories), University of the Western Cape, Robert Sobukwe Road, Bellville 7535, Cape Town, South Africa; mmasikini@uwc.ac.za (M.M.); 3438587@myuwc.ac.za (P.M.); 3693152@myuwc.ac.za (M.E.R.); 2810366@myuwc.ac.za (S.D.)

**Keywords:** band gap, chalcogenides, copper zinc tin sulfide (CZTS), copper zinc tin sulfide telluride (CZTSTe), kesterite, small angle X-ray scattering (SAXS)

## Abstract

Metal chalcogenides such as copper zinc tin sulfide (CZTS) have been intensively studied as potential photovoltaic cell materials, but their viability have been marred by crystal defects and low open circuit potential (*V*_oc_) deficit, which affected their energy conversion efficiency. Strategies to improve on the properties of this material such as alloying with other elements have been explored and have yielded promising results. Here, we report the synthesis of CZTS and the partial substitution of S with Te via anion hot injection synthesis method to form a solid solution of a novel kesterite nanomaterial, namely, copper zinc tin sulfide telluride (CZTSTe). Particle-size analyzed via small angle X-ray scattering spectroscopy (SAXS) confirmed that CZTS and CZTSTe materials are nanostructured. Crystal planes values of 112, 200, 220 and 312 corresponding to the kesterite phase with tetragonal modification were revealed by the X-ray diffraction (XRD) spectroscopic analysis of CZTS and CZTSTe. The Raman spectroscopy confirmed the shifts at 281 cm^−1^ and 347 cm^−1^ for CZTS, and 124 cm^−1^, 149 cm^−1^ and 318 cm^−1^ for CZTSTe. High degradation rate and the production of hot electrons are very detrimental to the lifespan of photovoltaic cell (PVC) devices, and thus it is important to have PVC absorber layer materials that are thermally stable. Thermogravimetric analysis (TGA) analysis indicated a 10% improvement in the thermal stability of CZTSTe compared to CZTS at 650 °C. With improved electrical conductivity, low charge transfer resistance (*R*_ct_) and absorption in the visible region with a low bandgap energy (*E*_g_) of 1.54 eV, the novel CZTSTe nanomaterials displayed favorable properties for photovoltaics application.

## 1. Introduction

The use of kesterite, which is a metal chalcogenide (copper zinc tin sulfide, CZTS), as in the development of the absorber layer of a solar cell has been extensively explored [1,2]. The material has progressively shown viability as a potential alternative for crystalline silicon in thin-film solar cell applications [3,4]. Kesterite has the advantage of being composed of naturally occurring abundant and non-toxic elements such as copper (Cu), zinc (Zn), tin (Sn) and sulfur (S). It also has the advantage of being a direct bandgap material with a band gap energy (*E*_g_) value of 1.5 eV, which is optimal for semiconductor materials for photovoltaic (PV) application [1]. Despite the interest in kesterites, its low power conversion efficiency (PCE) value of 11.0% has hampered its full-scale industrial production for solar cell application [5]. Much research effort has focused on finding ways to improve on the properties of the material [6]. Among the ideas explored, is the substitution of the constituent elements of the conventional kesterite material [7,8,9]. With regard to the cation components, Cu, Zn and Sn have been either fully or partially substituted with other elements [9]. This has led to improvement in the properties of the material for photovoltaic application, but the 15% PCE needed for industrial-scale production is yet to be achieved [10]. The anion component of kesterite, sulfide, has also been substituted by members of the chalcogen group (i.e. group 16) of the periodic table of elements. Selenium, which has been used to completely replace S to form kesterite material composed of copper zinc tin selenide (CZTSe), has recorded a PCE value of 11.8% [11,12,13]. Partial substitution of S in kesterite material by Se, giving a solid solution of copper zinc tin sulfide selenide (CZTSSe), has so far recorded the highest efficiency obtained for kesterite materials, with a PCE value of 12.6% [14]. This higher efficiency obtained for the solid solution of CZTSSe is related to the change in the *E*_g_ of the material which is brought about by the change in the anion species [15]. The *E*_g_ of the solid solution has the advantage of matching the energy corresponding to the maximum efficiency in the Shockley–Queisser plot for a single absorber cell [15]. The tunability of the *E*_g_ of the material from 1.5 to 2.1 eV makes it suitable for constituent-element substitutions [16,17]. This makes the material suitablefor use as the uppermost component in tandem multijunction solar cell when substituted with elements such as silver (Ag) and germanium (Ge) [18,19]. In reported studies, a trend has been observed in the *E*_g_ values of the kesterite materials of chemical composition CZTX (where X = S, Se or Te). The *E*_g_ trend is as follows: *E*_g_(CZTS, 1.5 eV) > *E*_g_(CZTSe, 1.03 eV) > *E*_g_(CZTTe, 0.98 eV). This type of *E*_g_ trend has been observed in germanium-substituted kesterite materials (Cu_2_ZnGeX_4_) for which *E*_g_(Cu_2_ZnGeS_4_) > *E*_g_(Cu_2_ZnGeSe_4_) > *E*_g_(Cu_2_ZnGeTe_4_) [20]. However, the inclusion of more than one chalcogenide in kesterites to form solid solutions lowers the *E*_g_ value. For example, *E*_g_(CZTS, 1.5 eV) > *E*_g_(CZTSSe, 1.13 eV) [19]. 

Tellurium has been used specifically in the solar cell industry due to its metalloid and semiconductor properties [21,22]; for example, cadmium telluride solar cell, which is currently produced on a large scale [23,24]. This study explores the formation of a novel solid solution composed of copper zinc tin sulfide telluride (CZTSTe), in which Te is incorporated into the kesterite structure to improve the properties of the material for possible photovoltaic cell application. The novel CZTSTe kesterite material was compared with pristine CZTS to demonstrate how the inclusion of Te improved the properties of the kesterite. A small amount of Te was used in comparison to S, taking into consideration the earth abundance of the constituent elements (Cu, Zn, Sn and S) of CZTS kesterite material, so as to minimize the cost of production and toxicity [1,25]. Various methods have been used in the synthesis kesterite materials such as evaporation and sputtering of the elements. The solution process has emerged as the most versatile method of synthesis, having advantage for industrial-scaled production of solar cells on glass or polymer substrates [26]. The best efficiency (PCE value of 12.6%) reported for a kesterite solar cell was for CZTSSe, which was produced via the solution process [19]. This process involved the use of metal-hydrazine as metal precursor source, but it has the demerit of being harmful and difficult to handle [27]. Other non-harmful solution processes have been reported in the synthesis of kesterite materials, including hot injection method [28]. The hot injection method was explored in this work for the synthesis of the kesterite nanoparticles.

## 2. Materials and Methods

### 2.1. Materials

All the reagents were of analytical grade and no further purification was performed. The following reagents used were purchased from Sigma-Aldrich, Johannesburg, South Africa: copper (II) chloride (anhydrous, powder, ≥99.995% trace metals basis), zinc chloride (anhydrous, powder, ≥99.995% trace metals basis), tin (II) chloride (anhydrous, powder, ≥99.99% trace metals basis), isopropanol (American Chemical Society, ACS, reagent grade, ≥99.5%), diethylene glycol (analytical grade), sodium borohydride (granular, 99.99% trace metals basis), Te powder (powder, 75 maximum particle size (micron), purity 99.5%), and S powder (powder, 99.98% trace metals basis).

### 2.2. Synthesis

This work reports a modified hot-injection technique of Dong *et al*. [29] for the synthesis of nanostructured materials. All experiments were performed under inert condition. The hot-injection method involved the preparation of two precursors which were later mixed. A total of 1.68 mmol of copper (II) chloride, 1.1 mmol of zinc chloride, and 1.0 mmol tin chloride were added to 120 mL of diethylene glycol (DEG) in a two-necked flask and was refluxed at one of the necks, which performed two roles: (a) inlet for the inert gas and (b) inlet and outlet for water throughout the experiment. The mixture was allowed to reach a temperature of 80 °C. This mixture made up the first precursor (cation precursor). For the second precursor (anion precursor), elemental S and Te powder with a total of 3.78 mmol were added to 10 mL of diethylene glycol and 7.36 mmol of sodium borohydride. The S:Te molar ratio in the mixture was 3:1. The mixture was heated slowly above 60 °C to facilitate the reduction process, which was confirmed by color change for both S and Te. When the temperature of the cation precursor had reached 80 °C, the anion precursor solution was gradually injected into reaction vessel containing the cation precursor under magnetic stirring, leading to a color change from yellowish brown to deep black, indicating initial and increased formation of the kesterite nanocrystals had occurred. The new mixture was then allowed to heat up to 190 °C and allowed to stand for 30 min and was subsequently naturally cooled to room temperature. After the cooling process, the nanoparticles were precipitated by diluting with isopropanol followed by 10 min of centrifugation at 4500 rpm. This process of washing and centrifuging was repeated twice, and the precipitates were air-dried. The nanoparticles were dispersed in isopropanol and lithium perchlorate for subsequent characterizations.

### 2.3. Instruments

Absorption studies in the wavelength region of 300–900 nm at room temperature were observed via Varian Cary 300 Ultraviolet-Visible- Near-infrared (UV-Vis–NIR) Spectrophotometer (Agilent, Santa Clara, CA, USA). X-ray diffraction (XRD) analysis was performed using a Philips PW 1830 X-ray diffractometer (Philips, Amsterdam, The Netherlands) with Cu Kα radiation at wavelength of 1.5406 Å. MATCH and DIAMOND softwares (Crystal Impact, Bonn, Germany) were used in analyzing the XRD data, as well as the simulation of the crystal structures of the synthesized nanoparticles. Simultaneous thermogravimetric analysis (TGA) and differential thermal analysis (DTA) analyses were performed with a TGA 4000 Thermogravimetric Analyzer from Perkin-Elmer (Boston, MA, USA). The TGA/DTA measurements were performed at a temperature range of 30 - 600 °C under nitrogen gas atmosphere and a heating rate of 10 °C min^−1^ with a sample size of 1 mg. The structure of the synthesized materials was studied with a Spectrum II Perkin-Elmer Fourier Transform Infrared (FTIR) Spectrometer (Perkin-Elmer, Boston, MA, USA) for 400 - 4500 cm^−1^. An Auriga field emission scanning electron microscope (FESEM) that was fitted with an energy-dispersive X-ray spectrometer (EDS) (Carl Zeiss Microscopy GmbH, Jena, Germany) and operated at an acceleration voltage of 200 kV, was used for morphological and elemental and morphological analyses of samples. A Field Electron and Ion Company (FEI) Tecnai G2 F20X-Twin MAT 200 kV Field Emission Transmission Electron Microscope (Eindhoven, The Netherlands) with energy-dispersive X-ray spectrometry (EDS) and selected area electron diffraction (SAED) capabilities, was used to perform high-resolution transmission electron microscopy (HR-TEM) experiments. All the electrochemical data were obtained with PalmSens 4 (PalmSens BV, Houten, The Netherlands), which was connected to a 0.071 cm^2^ glassy carbon disk working electrode, platinum wire (Pt) counter electrode and silver–silver chloride (3 M NaCl salt bridge type) reference electrode in a 10 mL electrochemical cell. Electrochemical analysis of each of the nanoparticles (CZTS or CZTSTe) was performed in 3 mL 0.1 M tetrabutylammonium phosphate-lithium perchlorate (TBAP-LiClO_4_) electrolyte.

## 3. Results and Discussion

The discussion of the experimental results of CZTS and CZTSTe nanomaterials are categorized into two. The first section (Section 3.1) involves the characterization studies used to investigate the overall composition of the nanomaterials. The second section (Section 3.2) deals with the comparison of the properties of CZTS and CZTSTe nanomaterials. 

### 3.1. Characterisation Studies

#### 3.1.1. Morphological Analysis

**Figure 1:**
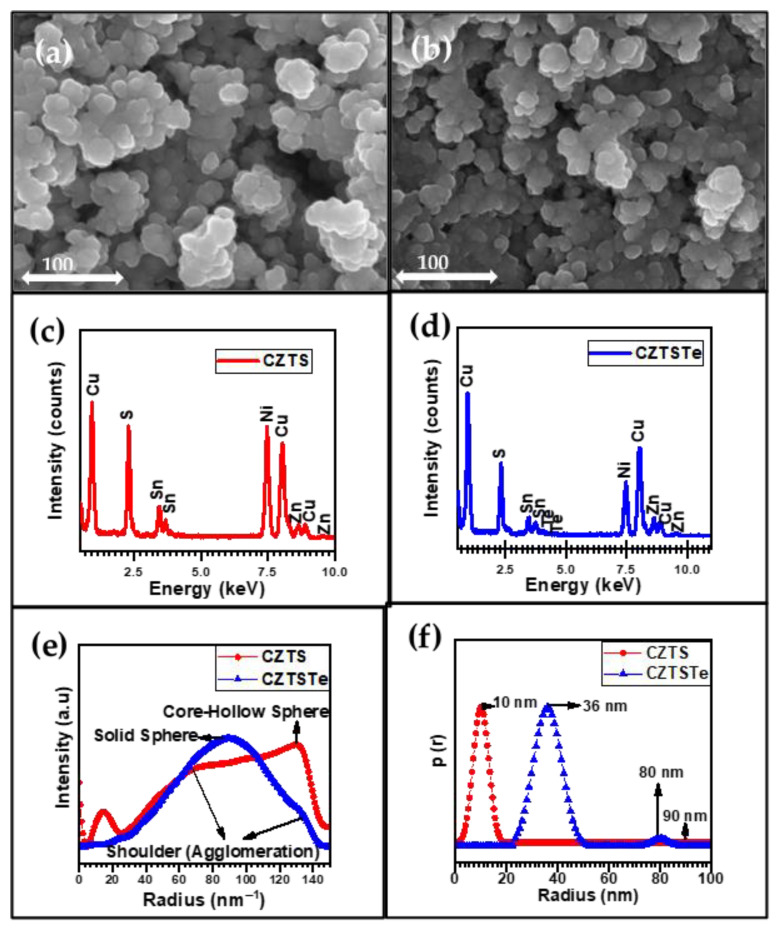
SEM micrograph of (**a**) copper zinc tin sulfide (CZTS) and (**b**) copper zinc tin sulfide telluride (CZTSTe). Energy-dispersive X-ray spectrometer (EDS) spectra of (**c**) CZTS and (**d**) CZTSTe. Small-angle X-ray scattering (SAXS) spectra for (**e**) shape and (**f**) size plots {p(r) represents distance distribution function}.

In the synthesis of nanoparticles, it is important to control the growth and dispersity [30]. Emphasis is laid on the shape of the nanomaterials, as it determines properties and reactivity [30]. Scanning electron microscopy (SEM) was used to evaluate the size distribution and shape of CZTS and CZTSTe nanomaterials. The SEM micrographs in Figure 1a,b which are for CZTS and CZTSTe, respectively, indicate morphological homogeneity of the nanoparticles. Spherically shaped particle images can be seen for both nanomaterials with uniform size distribution. Small-sized nanoparticles were formed by the two type of nanomaterials, which formed clusters from the agglomeration of particles. The agglomerated nanoparticles gave a distinctive flower-like morphology. Previous reports have accepted that the flower-like morphology is associated with good photocatalytic properties in kesterite nanomaterials [29]. Electron-dispersive X-ray spectroscopy (EDS) analysis gave the chemical composition of the nanomaterials of CZTS and CZTSTe as presented in Figure 1c,d, respectively. Observable nickel peaks emanated from the use of nickel grid as the sample holder. The difference in the S content of both nanomaterials was revealed in the EDS spectra. There was an increased intensity in the S signal in CZTS compared to CZTSTe, as shown in Figure 1c,d, respectively. This is because some of the atoms of S are replaced by Te to form the CZTSTe. Moreover, the successful inclusion of Te into the kesterite structure is evidenced by the presence of the Te peaks in the spectra of CZTSTe, as can be seen in Figure 1d. The average atom content for both nanoparticles is summarized as Cu_1.92_Zn_1.0_Sn_0.8_S_4.26_ for CZTS and Cu_1.91_Zn_1.0_Sn_0.64_(S_0.84_,Te_0.47_)_4_ for CZTSTe. These chemical compositions were in close conformity to the general chemical composition of Cu_2_ZnSnS_4_ for CZTS and Cu_2_ZnSn(S_0.75_,Te_0.25_)_4_ for CZTSTe [31].

Significant morphological data can be obtained by utilizing the small-angle X-ray scattering (SAXS) technique [32,33]. SAXS measurement provides general spectra of the sizes and shapes of the nanoparticles [33]. The SAXS spectra plotted in Figure 1e shows solid sphere shape for CZTSTe nanoparticles and a core-hollow sphere shape for CZTS nanoparticles. These underlying spherical shapes obtained for both nanomaterials agree with the spherical shape obtained in SEM micrographs in Figure 1a,b. The spectra of CZTSTe in Figure 1e revealed the presence of shoulders which is indicative of the agglomeration of nanoparticles. The average size distribution of CZTS nanoparticles is 10 nm, while that of CZTSTe is 36 nm (see Figure 1f). The increase in the size of CZTSTe can be attributed to the inclusion of large Te atom in the kesterite material. The figure indicates that the CZTSTe sample also contains particles of larger sizes (~80 nm), which can be attributed to agglomeration. Since CZTSTe particles are still nanostructured (<100 nm) in agglomerated and non-agglomerated morphologies, CZTSTe is expected to exhibit excellent photophysical properties [34].

**Figure 2:**
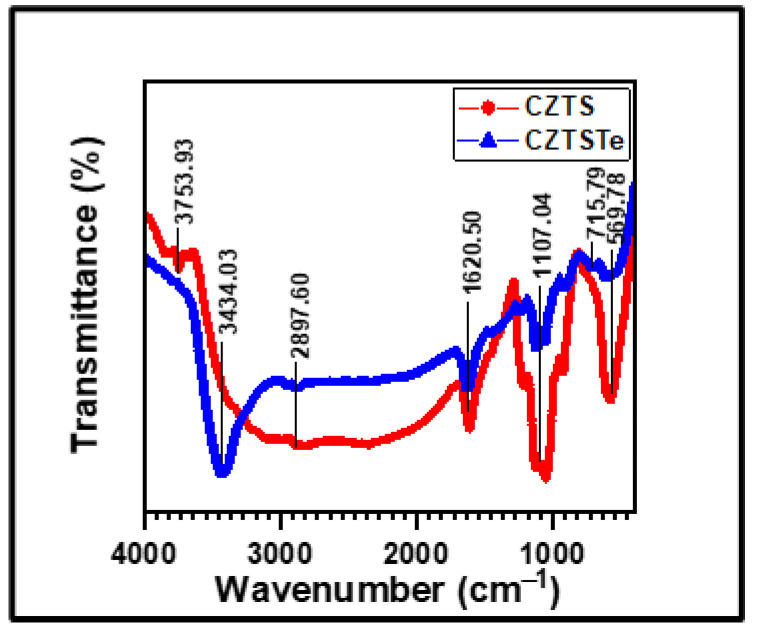
FTIR spectrum of CZTS and CZTSTe nanomaterials.

Fourier transform infrared spectroscopy (FTIR) was used for the determination of bonding characteristics of the kesterite nanomaterials [32]. The vibrational bands in Figure 2 were assigned as follows: *ν*(O–H) 3753.93 cm^−1^) [29], *ν*(C–H) 2897.60 cm^−1^ [29], *ν*(C–C) 1620.50 cm^−1^ [29], *ν*(C–O) 1107.04 cm^−1^ [29,32] and *ν*(S–S) 569.78 cm^−1^ [35,36]. CZTSTe showed similar vibrational bands and they were assigned as *ν*(O–H) 3434.03 cm^−1^, *ν*(C–H) 2897.60 cm^−1^, *ν*(C–C) 1620.50 cm^−1^, *ν*(C–O) 1107.04 cm^−1^, *ν*(Te=O) 715.79 cm^−1^ [36] and *ν*(S–S) 569.78 cm^−1^. The presence of S in both nanomaterials can be seen from the vibrational band at 569.78 cm^−1^. The Te=O vibrational band at 715.79 cm^−1^ in the spectrum of CZTSTe, confirms the successful integration of Te into the structure of the newly synthesized nanomaterial. The vibration modes of the organic functional groups the spectra are due to the remnants of the solvents (diethylene glycol and isopropanol) used for synthesis [29]. The solvents are easily removed by annealing at high temperatures of about 400–500 °C during device fabrication.

#### 3.1.2. Crystal Analysis

**Figure 3:**
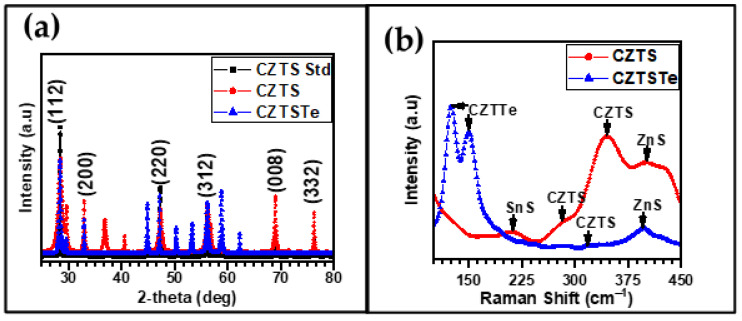
(**a**) XRD and (**b**) Raman spectra of CZTS and CZTSTe nanomaterials.

**Figure 4:**
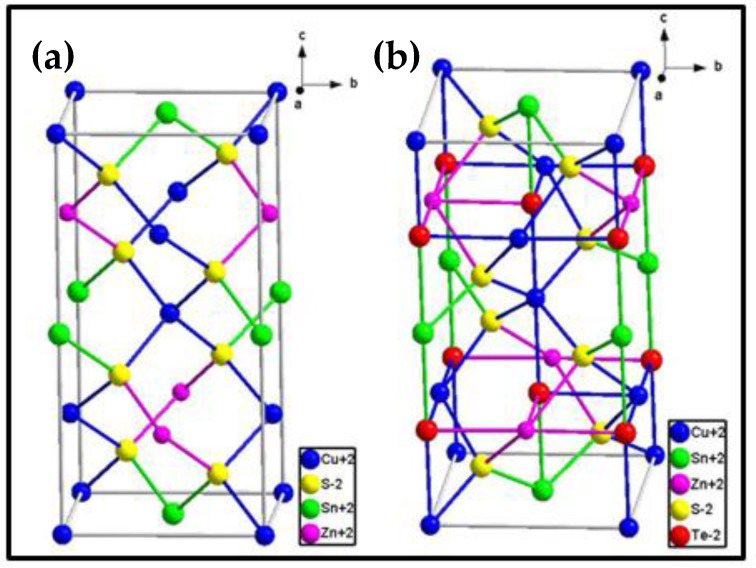
Simulated crystal structures of (**a**) CZTS and (**b**) CZTSTe (blue = Cu; green = Sn; pink = Zn; red = Te; yellow = S).

The most versatile technique employed for determining the crystal structure of materials is X-ray diffractometry (XRD). This spectroscopic method allows for the identification of chemical compounds from their crystal structure. Figure 3a shows the XRD patterns of the CZTS and CZTSTe nanomaterials. The patterns for CZTS displayed major peaks at *2θ* = 28.37°, 32.94°, 47.23° and 56.31°, which correspond to the crystal interplanar spacing or crystal lattice spacing (*d-spacing*) values of 3.1346, 2.7091, 1.9230 and 1.6406 Å, respectively. The patterns for CZTSTe had major peaks at *2θ* = 28.26°, 32.84°, 47.19° and 56.08°, with *d*-*spacing* values of 3.1330, 2.7072, 1.9225 and 1.6379 Å, respectively. These peaks were identified using Joint Committee on Powder Diffraction Standards (JCPDS) card no. of 00-026-0575. These peaks corresponded to the 112, 200, 220 and 312 planes of the kesterite phase, respectively, which has a tetragonal modification [37]. Conventionally, CZTS can assume different crystal-phase modifications such as kesterite, stannite and wurtzite [38,39]. The kesterite phase is the most suitable for the materials due to the high stability of the phase. The crystal lattice parameters *a*, *b* and *c* (i.e. the lengths of the 3 edges of the crystal unit cell) and were *α, β* and *γ* (the angles between the crystal unit cell edges) were evaluated for the nanomaterials. The values obtained for CZTS were *a = b =* 5.43682 Å and *c =* 10.8388 Å, while CZTSTe had values of *a = b =* 5.44221 Å and *c =* 10.8578 Å. The *α, β* and *γ* values were 90° for CZTS and CZTSTe. These values of the lattice parameters are in good agreement with values for the tetragonal kesterite phase for which *a = b =* 5.430 Å and *c =* 10.8680 Å [40]. The simulated CZTS and CZTSTe crystal structures are shown in Figure 4. The simulated crystal structure of CZTSTe shows the average bonding of one Te atom to two Cu atoms, one Zn atom and one Sn atom, which is the typical type of bonding found in chalcogenides. 

XRD data can provides useful the information for estimating the crystal size of materials. For this purpose, the Debye–Scherrer equation (Equation (1)), was used to calculate the crystal size of the materials:

(1) $$D=\frac{k\lambda }{\beta \cos\theta }$$

In Equation (1) *D* is the mean size of the crystal in Å; *k* is a dimensionless shape factor with value of ~ 0.9; *λ* is the X-ray wavelength; *β* is the line broadening, i.e. the full width at half maximum (FWHM) of the peak in radians; and *θ* is the Bragg angle in radians. The calculated crystallite size for the most prominent peak (112 planes) of both nanomaterials was 106.6 Å (~11 nm) for CZTS and 310.2 Å (31 nm) for CZTSTe. Parameters used for calculating the crystal size are summarized in Table 1 below. The particle sizes values calculated from the XRD data are in good agreement with those obtained with SAXS.

Earlier studies have shown that XRD is not enough to categorically establish the presence of the desired kesterite phase, as its characteristic peaks appear at the same peak position (the same *2θ* value) as binary, secondary and ternary phases, such as for CuS, ZnS and Cu_2_SnS_3_ [10]. Raman spectroscopy was used to confirm the kesterite phase and establish the absence of secondary phase formation in CZTSTe [29]. Figure 3b shows the Raman spectra of CZTS and CZTSTe. The spectrum for CZTS has peaks at 212 cm^−1^, 281 cm^−1^, 347 cm^−1^ and 400 cm^−1^, which are attributed to SnS, CZTS, CZTS, and ZnS, respectively [29]. CZTSTe has peaks at 124 cm^−1^, 149 cm^−1^, 318 cm^−1^ and 395 cm^−1^, which originated from CZTTe, CZTTe, CZTS and ZnS, respectively [41]. Although the Raman spectra confirmed the presence of the kesterite phase in both nanomaterials, secondary phases such as SnS and ZnS (in CZTS) and ZnS (in CZTSTe) were present. Notably, the peaks for the kesterite phase were more prominent in the two nanomaterials. 

**Figure 5:**
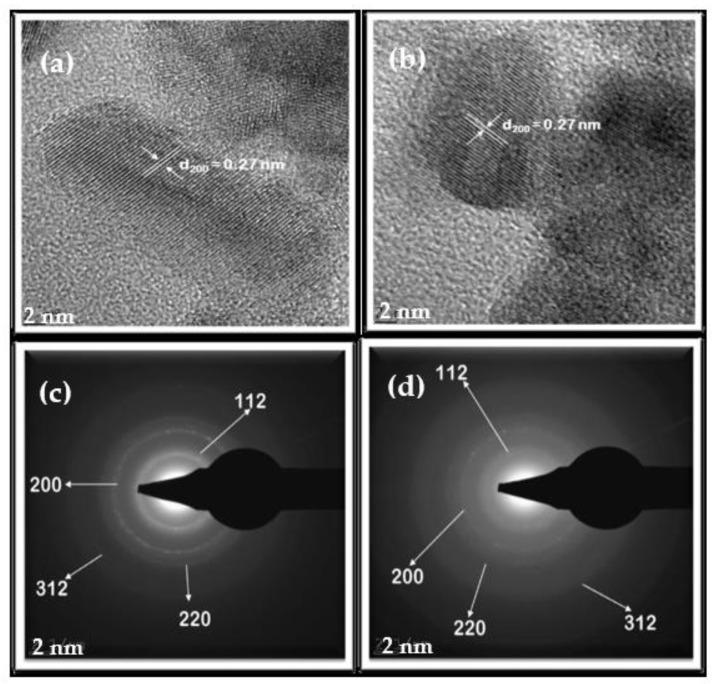
High-resolution transmission electron microscopy (HRTEM) micrographs showing lattice fringes for (**a**) CZTS and (**b**) CZTSTe. The selected area electron diffraction (SAED) images for (**c**) CZTS and (**d**) CZTSTe.

Selected-area electron diffraction (SAED) is a useful technique for structural and crystal defects analyses. Monocrystalline materials give rise to SAED micrographs that have distinct spots and well-defined lattice fringes corresponding to specific planes [32]. The SAED micrographs of polycrystalline nanomaterials, on the other hand, display ring patterns consisting of discrete spots formed by crystals of different orientations (i.e. that have different planes) [32]. High-resolution transmission electron microscopy (HRTEM) micrographs and the SAED pattern of CZTS and CZTSTe are presented in Figure 5. Lattice fringe analysis of the HRTEM images of CZTS and CZTSTe in Figure 5a and CZTSTe Figure 5b, respectively, gave a *d-spacing* value of ~ 0.27 nm. This corresponds to the 200 plane of the kesterite phase, as was also shown in the XRD spectra. The SAED images shown in Figure 5c (for CZTS) and Figure 5d (for CZTSTe) display polycrystalline concentric ring patterns that correspond to the 112, 200, 220 and 312 crystal planes, thereby validating the XRD results presented in Figure 3a [29].

### 3.2. Comparative Studies

#### 3.2.1. Stability Analysis

**Figure 6:**
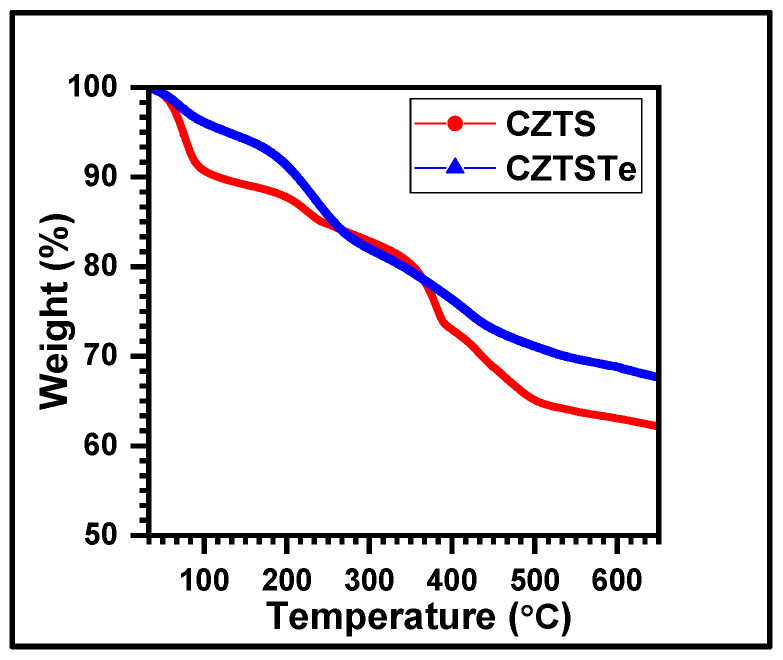
Thermogravimetric analysis (TGA) spectra of CZTS and CZTSTe nanoparticles.

Figure 6 presents the thermogravimetric analysis (TGA) data of CZTS and CZTSTe for the temperature range of 30–650 °C at a heating rate of 10 °C min^−1^ in argon atmosphere. The initial weight loss of about 9% for CZTS and 4% for CZTSTe at about 100 °C, is attributed to the evaporation of water. Further 4% weight loss for CZTS and 5% for CZTSTe at about 200 °C is due to the evaporation of isopropanol and diethylene glycol which were synthesis solvents. Further heating to 400 °C resulted in a ~14% weight decrease for CZTS and ~13% for CZTSTe due to the loss of superficial chalcogens. The loss of Sn by the sublimation of SnS at temperatures above 400 °C, caused the weight to decrease by 9% and 8% for CZTS and CZTSTe, respectively. These finding are similar to the pattern reported in previous studies [29]. The TGA spectra show that at 650 °C, CZTS and CZTSTe exhibit 62% and 68% retention of their original weights, respectively. This indicates that the replacement of some S atoms with Te improved the thermal stability of the kesterite material. This is because Te (*MP* = 449.51 °C, *BP* = 988 °C and *κ* = 3 W/mK) is more thermally stable than S (*MP* = 115.21 °C, *BP* = 444.72 °C and *κ* = 0.205 W/mK) [42,43,44]; where *MP*, *BP* and *κ* are melting point, boiling point and thermal conductivity, respectively. 

#### 3.2.2. Optical Analysis

**Figure 7:**
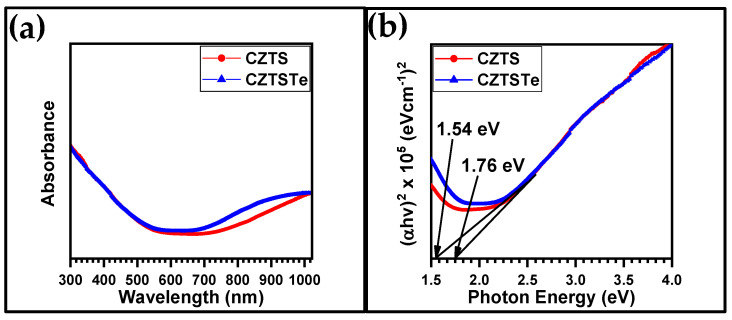
(**a**) UV–Vis absorption spectra and (**b**) Tauc plots for CZTS and CZTSTe nanomaterials.

Ultraviolet visible spectroscopy (UV–Vis) technique was used to study the absorption properties of the nanoparticles. The spectra obtained are plotted in Figure 7a.

For direct band gap materials such as kesterites, the Tauc plot (i.e. (*αhν*)^2^ vs. *hν*, where *hν* is the photon energy and *α* is the absorption coefficient of the material, is used to determine their bandgap (*E*_g_) values. The Tauc plots for CZTA and CZTSTe are presented in Figure 7b and the corresponding *E*_g_ values are 1.76 eV and 1.54 eV, respectively. The incorporation of Te to kesterite nanomaterial narrowed the band gap towards the optimum *E*_g_ value (1.5 eV) required for solar cell application. It has been reported that a high S content in kesterite increases the *E*_g_ value, due to its insulating properties [43]. The low *E*_g_ value obtained by the inclusion of Te in the kesterite, implies that it allows very good absorption of the visible light as required for photovoltaic application [32]. Other workers have also reported the reduction of *E*_g_ value by the addition of Te and other metal chalcogenides into the kesterite structure [20,41,42,43,44,45]. This is because Te is a semiconductor and has higher thermal conductivity than S [46]. Also, the reduction in the *E*_g_ value obtained for CZTSTe nanomaterial, was due to quantum confinement effects which increases the absorption wavelength [47,48]. Other reports [49,50,51] have shown that for polydispersed nanomaterials, such as CZTS and CZTSTe (as shown in Figure 1e,f), the large nanoparticles exhibit redshift absorptions while the small ones give blueshifts. CZTS formed 10 nm particles and 90 nm clusters, while CZTSTe formed 36 nm particles and 80 nm clusters. The clusters induced a redshift absorption, as supported by the quantum confinement theory of nanoparticulated materials [47], thus making them suitable for light-emitting, photocatalytic and photovoltaic applications. The formation of clusters can also enhance the conductivity of the nanomaterials as a result of percolation effect [52,53,54]. 

#### 3.2.3. Electrochemical Analysis

**Figure 8:**
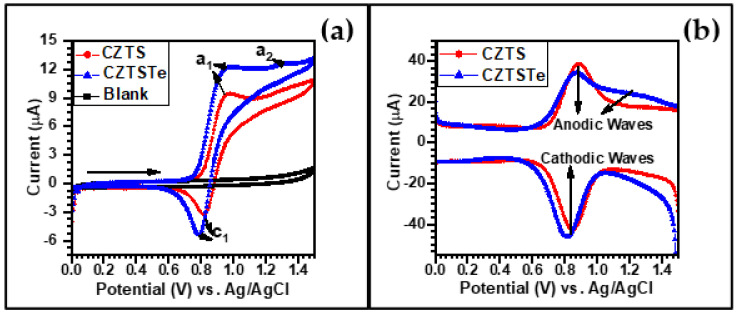
(**a**) Cyclic voltammograms obtained at 10 mV s^−1^ and (**b**) square-wave voltammograms of 4 mM CZTS and 4 mM CZTSTe in 0.1 M lithium perchlorate in acetonitrile.

Figure 8a shows the cyclic voltammograms (CVs) of CZTS, CZTSTe and the electrolyte at scan rate of 10 mV s^−1^. The CVs of CZTS and CZTSTe portray one distinctive redox couple, i.e. anodic (**a_1_**) and cathodic (**c_1_**) peaks, and an addition anodic peak (**a_2_**) for CZTSTe. The anodic peak potential (*E**_pa_***) values for CZTSTe are 0.97 V and 1.32 V for **a_1_** and **a_2_**, respectively; while the cathodic peak potential (*E**_pc_***) value is 0.79 V for **c_1_**. The *E**_pa_*** values of 0.97 V corresponds to the oxidation of copper [55,56] and that of 1.32 V (i.e. **a_2_**) corresponds to the oxidation of Te^2-^ to Te(IV) [57,58]. The *E**_pc_*** values of 0.79 V is attributed to the reduction of copper. For CZTS *E**_pa_*** and *E**_pc_*** values are 0.96 V (for **a_1_**) and 0.82 V (for **c_1_**), respectively, and refer to the oxidation and reduction of Cu. The value of the anodic peak current/cathodic peak current, *I**_pa_***(**a_1_**)/*I**_pc_***(**c_1_**), is ~1 for both materials, which indicates a reversible fast electron transfer process for the Cu^+^/Cu^2+^ electrochemistry. However, the formal potential, *E**^0^***′ = (*E**_pa_*** + *E**_pc_***)/2, values for CZTS and CZTSTe are 0.89 V and 0.88 V, respectively, and the currents density of CZTSTe is by a factor of 1.3 larger than that of CZTS, showing an improvement in the rate of the charge transfer reaction, which is possibly, due to the inclusion of Te in the kesterite [59,60]. 

The square-wave voltammetry (SWV) was used to study the electrochemical stability of the materials [61]. The results obtained for the nanomaterials are plotted in Figure 8b. For CZTS, the peak potentials for the anodic and cathodic square waves are the same, and their peak current values are the same, implying that the material is electrochemically stable under the experimental conditions. However, for CZTSTe the values of the peak potential and current are larger for the cathodic waves than for the anodic wave. This increase in the peak values for the cathodic wave means that the reduction of Cu^2+^ to Cu^+^ (as discussed for Figure 8a) is coupled to another process, namely the reduction of Te(IV) to Te^2−.^

**Figure 9:**
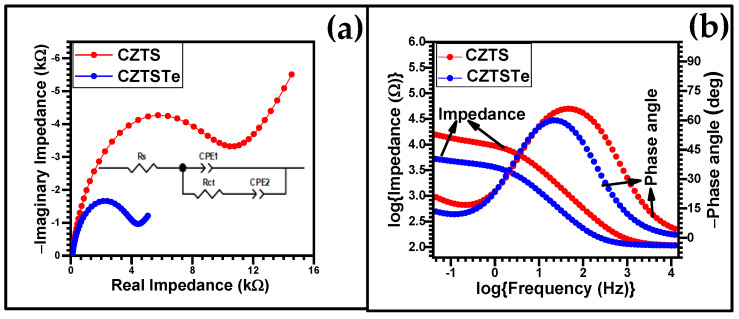
Electrochemical impedance spectroscopy (EIS) data: (**a**) Nyquist and (**b**) Bode plots for CZTS and CZTSTe nanomaterials. (0.1 M lithium perchlorate in acetonitrile was used as the supporting electrolyte).

The electrochemical impedance spectroscopy (EIS) data of CZTS and CZTSTe are presented in Figure 9a as Nyquist plots. The Randle’s equivalent circuit (Figure 9a insert) was used to fit the data. In the circuit diagram, Solution resistance (*R*_s_ is solution resistance, constant phase element is the CPE and *R*_ct_ is charge transfer resistance. The values obtained for the parameters as written in Table 2. The *R*_ct_ values obtained for CZTSTe and CZTS are 3.9 kΩ and 9.8 kΩ, respectively, implying greater conductivity properties of CZTSTe. The Bode −phase angle (−*θ*) and log (total impedance), log *Z*, plots of the EIS data are contained in the Figure 9b. The *Z* value calculated for CZTS (16.6 kΩ) is significantly higher than that of CZTSTe (5.5 kΩ). The −*θ*_peak_ values of CZTS (67.5°) and CZTSTe (60°) confirm that both materials are semiconductors [50,51]. However, the peak frequency (*ν*_peak_) values are CZTS (45.71 Hz) and CZTSTe (21.38 Hz). The lower *ν*_peak_ value for CZTSTe indicates its greater propensity for interfacial faster charge transfer reaction compared to CZTS.

The *R*_ct_, *ν*_peak_ and Z values obtained from both the Nyquist and the Bode analyses, point to an improved electro-conductivity of CZTSTe compared to CZTS. A similar trend was observed in the CV analysis of the materials, where the cathodic and anodic peak current densities of CZTS were by a factor of 1.3 lower than those of CZTSTe, due to the greater conductivity of the Te-containing material. Literature antecedents also, confirmed that the conductivity of metal chalcogenide materials improved as elements down the group 16 of the periodic table are incorporated in the material, with Te being the most effective [45]. 

## 4. Conclusions

A novel CZTSTe nanostructed kesterite material was successfully synthesized through the anion hot injection method, which is a non-toxic solid solution process. The size, shape and structure of the material were confirmed with SAXS, SEM, TEM, Raman and XRD spectroscopy experiments. The incorporation of Te in the CZTSTe nanomaterial improved the band gap energy, electro-conductivity, rate of charge transfer reaction and thermal stability; and brought about a redshift in its absorption characteristics. The improvement in the photophysical properties implies that the novel kesterite material holds much promise as an excellent material for solar cell application. The improved electrochemical impedance spectroscopy characteristics of CZTSTe, as deduced from the values of its Bode-phase angle and Bode-total impedance parameters, make it a very viable material for photovoltaic and photocatalytic applications.

## Figures and Tables

**Table 1:** Crystal parameters.

Material	*k*	*λ* (Å)	*β* (rad)	*θ* (rad)	*D* (Å)
CZTS	0.89	1.54058	0.0130	0.2483	106.6
CZTSTe	0.89	1.54058	0.00442	0.2484	310.2

**Table 2:** EIS parameters of CZTS and CZTSTe nanomaterials.

Sample	*R*_ct_ (kΩ)	*R*_s_ (kΩ)	−*θ*_peak_ (°)	*ν*_peak_ (Hz_)_	*Z* (kΩ)
CZTS	9.8	0.106	67.5	45.71	16.6
CZTSTe	3.9	0.107	60.0	21.38	5.5

## Data Availability

Data available on demand from corresponding authors. The data are not publicly available due to privacy restriction.

## References

[1] Giraldo S., Placidi M., Saucedo E. (2019). Kesterite: New Progress Toward Earth-Abundant Thin-Film Photovoltaic. Advanced Micro- and Nanomaterials for Photovoltaics.

[2] Siebentritt S., Schorr S. (2012). Kesterites-a challenging material for solar cells. Prog. Photovolt. Res. Appl..

[3] Repins I., Vora N., Beall C., Wei S.H., Yan F., Romero M., Teeter G., Du H., To B., Young M. Kesterites and chalcopyrites: A comparison of close cousins. Proceedings of the Materials Research Society Symposium Proceedings.

[4] Ramasamy K., Malik M.A., O’Brien P. (2012). Routes to copper zinc tin sulfide Cu2ZnSnS4 a potential material for solar cells. Chem. Commun..

[5] Todorov T.K., Reuter K.B., Mitzi D.B. (2010). High-efficiency solar cell with earth-abundant liquid-processed absorber. Adv. Mater..

[6] Mitzi D.B., Gunawan O., Todorov T.K., Wang K., Guha S. (2011). The path towards a high-performance solution-processed kesterite solar cell. Sol. Energy Mater. Sol. Cells.

[7] Su Z., Tan J.M.R., Li X., Zeng X., Batabyal S.K., Wong L.H. (2015). Cation Substitution of Solution-Processed Cu2ZnSnS4 Thin Film Solar Cell with over 9% Efficiency. Adv. Energy Mater..

[8] Lafond A., Guillot-Deudon C., Vidal J., Paris M., La C., Jobic S. (2017). Substitution of Li for Cu in Cu2ZnSnS4: Toward Wide Band Gap Absorbers with Low Cation Disorder for Thin Film Solar Cells. Inorg. Chem..

[9] Dhawale D.S., Ali A., Lokhande A.C. (2019). Impact of various dopant elements on the properties of kesterite compounds for solar cell applications: A status review. Sustain. Energy Fuels.

[10] Siebentritt S. (2013). Why are kesterite solar cells not 20% efficient?. Thin Solid Film..

[11] Ilari G.M., Fella C.M., Ziegler C., Uhl A.R., Romanyuk Y.E., Tiwari A.N. (2012). Cu 2ZnSnSe 4 solar cell absorbers spin-coated from amine-containing ether solutions. Sol. Energy Mater. Sol. Cells.

[12] Walker B.C., Negash B.G., Szczepaniak S.M., Brew K.W., Agrawal R. CZTSe devices fabricated from CZTSSe nanoparticles. Proceedings of the Conference Record of the IEEE Photovoltaic Specialists Conference.

[13] Volobujeva O., Raudoja J., Mellikov E., Grossberg M., Bereznev S., Traksmaa R. (2009). Cu2ZnSnSe4 films by selenization of Sn-Zn-Cu sequential films. J. Phys. Chem. Solids.

[14] Wang W., Winkler M.T., Gunawan O., Gokmen T., Todorov T.K., Zhu Y., Mitzi D.B. (2014). Device characteristics of CZTSSe thin-film solar cells with 12.6% efficiency. Adv. Energy Mater..

[15] Chen S., Gong X.G., Walsh A., Wei S.H. (2009). Crystal and electronic band structure of Cu2 ZnSn X4 (X=S and Se) photovoltaic absorbers: First-principles insights. Appl. Phys. Lett..

[16] Huang D., Persson C. (2013). Band gap change induced by defect complexes in Cu2ZnSnS4. Thin Solid Film..

[17] Podsiadlo S., Bialoglowski M., Fadaghi M., Matyszczak G., Kardas K., Dluzewski P., Data P., Lapkowski M. (2015). Synthesis of kesterite nanopowders with bandgap tuning ligands. Cryst. Res. Technol..

[18] Parasyuk O.V., Piskach L.V., Romanyuk Y.E., Olekseyuk I.D., Zaremba V.I., Pekhnyo V.I. (2005). Phase relations in the quasi-binary Cu_2_GeS_3_-ZnS and quasi-ternary Cu_2_S-Zn(Cd)S-GeS_2_ systems and crystal structure of Cu2ZnGeS4. J. Alloys Compd..

[19] Zhu T., Huhn W.P., Wessler G.C., Shin D., Saparov B., Mitzi D.B., Blum V. (2017). I2-II-IV-VI4 (I = Cu, Ag; II = Sr, Ba; IV = Ge, Sn; VI = S, Se): Chalcogenides for Thin-Film Photovoltaics. Chem. Mater..

[20] Chen D., Ravindra N.M. (2013). Electronic and optical properties of Cu2ZnGeX4 (X = S, Se and Te) quaternary semiconductors. J. Alloys Compd..

[21] Lin S., Li W., Chen Z., Shen J., Ge B., Pei Y. (2016). Te as a high-performance elemental thermoelectric. Nat. Commun..

[22] Chivers T., Laitinen R.S. (2015). Te: A maverick among the chalcogens. Chem. Soc. Rev..

[23] Lepiller C., Cowache P., Guillemoles J.F., Gibson N., Özsan E., Lincot D. (2000). Fast electrodeposition route for cadmium telluride solar cells. Thin Solid Film..

[24] Lee T.D., Ebong A.U. (2017). A review of thin film solar cell technologies and challenges. Renew. Sustain. Energy Rev..

[25] Babula P., Adam V., Opatrilova R., Zehnalek J., Havel L., Kizek R. (2008). Uncommon heavy metals, metalloids and their plant toxicity: A review. Environ. Chem. Lett..

[26] Zhang R., Szczepaniak S.M., Carter N.J., Handwerker C.A., Agrawal R. (2015). A versatile solution route to efficient Cu 2 ZnSn(S,Se) 4 thin-film solar cells. Chem. Mater..

[27] Van Embden J., Chesman A.S.R., Della Gaspera E., Duffy N.W., Watkins S.E., Jasieniak J.J. (2014). Cu2ZnSnS4 xSe4(1- x) solar cells from polar nanocrystal inks. J. Am. Chem. Soc..

[28] Todorov T., Hillhouse H.W., Aazou S., Sekkat Z., Vigil-Galán O., Deshmukh S.D., Agrawal R., Bourdais S., Valdés M., Arnou P. (2020). Solution-based synthesis of kesterite thin film semiconductors. J. Phys. Energy.

[29] Dong H., Schnabel T., Ahlswede E., Feldmann C. (2014). Polyol-mediated synthesis of Cu2ZnSn(S,Se)4 kesterite nanoparticles and their use in thin-film solar cells. Solid State Sci..

[30] Liu F., Wu J. (2010). Morphology Study by Using Scanning Electron Microscopy. Education.

[31] Kim D., Kim M., Shim J., Kim D., Choi W., Park Y.S., Choi Y., Lee J. (2016). Synthesis of CZTS Nanoparticles for Low-Cost Solar Cells. J. Nanosci. Nanotechnol..

[32] Sharma S.K., Verma D.S., Khan L.U., Kumar S., Khan S.B., Sharma S.K. (2018). Handbook of Materials Characterization.

[33] Schnablegger H., Singh Y. (2013). The SAXS Guide. Ant. Paar GmbH.

[34] Wang Z.L. (2001). Characterization of nanophase materials. Part. Part. Syst. Charact..

[35] Nakamoto K. (2008). Infrared and Raman Spectra of Inorganic and Coordination Compounds, Part A.

[36] Nakamoto K. (2008). Infrared and Raman Spectra of Inorganic and Coordination Compounds, Part B.

[37] Haass S.G., Diethelm M., Werner M., Bissig B., Romanyuk Y.E., Tiwari A.N. (2015). 11.2% Efficient Solution Processed Kesterite Solar Cell with a Low Voltage Deficit. Adv. Energy Mater..

[38] Regulacio M.D., Ye C., Lim S.H., Bosman M., Ye E., Chen S., Xu Q.H., Han M.Y. (2012). Colloidal nanocrystals of wurtzite-type Cu 2ZnSnS 4: Facile noninjection synthesis and formation mechanism. Chem. Eur. J..

[39] Yang W.C., Miskin C.K., Hages C.J., Hanley E.C., Handwerker C., Stach E.A., Agrawal R. (2014). Kesterite Cu2ZnSn(S,Se)4 absorbers converted from metastable, wurtzite-derived Cu2ZnSnS4 nanoparticles. Chem. Mater..

[40] Schorr S., Gonzalez-Aviles G. (2009). In-situ investigation of the structural phase transition in kesterite. Phys. Status Solidi Appl. Mater. Sci..

[41] Pareek D., Balasubramaniam K.R., Sharma P. (2016). Synthesis and characterization of kesterite Cu2ZnSnTe4: Via ball-milling of elemental powder precursors. RSC Adv..

[42] Gray T., Whitby M., Mann N. Technical Data for the Element Te in the Periodic Table. https://periodictable.com/Elements/052/data.html.

[43] Technical Data for the Element S in the Periodic Table. https://periodictable.com/Elements/016/data.html.

[44] Albury B.A. Why Thermal Conductivity Matters. http://www.puretemp.com/stories/why-thermal-conductivity-matters.

[45] Kumar S., Lal B., Aghamkar P., Husain M. (2009). Influence of S, selenium and Te doping on optical, electrical and structural properties of thin films of lead salts. J. Alloys Compd..

[46] Ilahi S., Almosni S., Chouchane F., Perrin M., Zelazna K., Yacoubi N., Kudrawiec R., Râle P., Lombez L., Guillemoles J.F. (2015). Optical absorption and thermal conductivity of GaAsPN absorbers grown on GaP in view of their use in multijunction solar cells. Sol. Energy Mater. Sol. Cells.

[47] Asha A.B., Narain R. (2020). Nanomaterials properties. Polymer Science and Nanotechnology.

[48] Roduner E. (2006). Size matters: Why nanomaterials are different. Chem. Soc. Rev..

[49] Link S., El-Sayed M.A. (1999). Spectral Properties and Relaxation Dynamics of Surface Plasmon Electronic Oscillations in Gold and Silver Nanodots and Nanorods. J. Phys. Chem. B.

[50] Mittal A.K., Banerjee U.C. (2016). Current status and future prospects of nanobiomaterials in drug delivery. Nanobiomaterials in Drug Delivery: Applications of Nanobiomaterials.

[51] Singh R., Soni R.K. (2018). Laser-Induced Heating Synthesis of Hybrid Nanoparticles. Noble Metal-Metal Oxide Hybrid Nanoparticles: Fundamentals and Applications.

[52] Stauffer D., Aharony A. (2003). Percolation. Encyclopedia of Physical Science and Technology.

[53] Mutiso R.M., Winey K.I. (2012). Electrical Conductivity of Polymer Nanocomposites. Polymer Science: A Comprehensive Reference, 10 Volume Set.

[54] Dang Z.M., Zheng M.S. (2018). Multiphase/multicomponent dielectric polymer materials with high permittivity and high breakdown strength. Dielectric Polymer Materials for High-Density Energy Storage.

[55] Aksu S., Doyle F.M. (2002). Electrochemistry of copper in aqueous ethylenediamine solutions. J. Electrochem. Soc..

[56] Teo W.Z., Ambrosi A., Pumera M. (2013). Direct electrochemistry of copper oxide nanoparticles in alkaline media. Electrochem. Commun..

[57] Bouroushian M. (2010). Electrochemistry of the Chalcogens BT—Electrochemistry of Metal Chalcogenides. Electrochem. Met. Chalcogenides Monogr. Electrochem..

[58] Bouroushian M. (2010). Electrochemistry of the Chalcogens. Electrochemistry of Metal Chalcogenides.

[59] Luque A., Martí A. (2010). Electron-phonon energy transfer in hot-carrier solar cells. Sol. Energy Mater. Sol. Cells.

[60] Man M.K.L., Margiolakis A., Deckoff-Jones S., Harada T., Wong E.L., Krishna M.B.M., Madéo J., Winchester A., Lei S., Vajtai R. (2017). Imaging the motion of electrons across semiconductor heterojunctions. Nat. Nanotechnol..

[61] Jadreško D., Zelić M. (2013). Cyclic multipulse voltammetric techniques. Part I: Kinetics of electrode processes. J. Electroanal. Chem..

